# Identifying novel amino acid substitutions of hemagglutinin involved in virulence enhancement in H7N9 virus strains

**DOI:** 10.1186/s12985-020-01464-1

**Published:** 2021-01-11

**Authors:** Ting Zhang, Haiwei Du, Li Guo, Feng Liu, Haoxiang Su, Fan Yang

**Affiliations:** MHC Key Laboratory of Systems Biology of Pathogen, Institute of Pathogen Biology, Chinese Academy of Medical Sciences and Peking Union Medical College, Beijing, China

**Keywords:** Amino acid substitutions, H7N9, Hemagglutinin

## Abstract

**Background:**

To identify site-specific features of amino acid substitutions that confer enhanced H7N9 virulence in humans, we independently generated mammalian-adapted variants of A/Anhui/1/2013 (AH-H7N9) and A/Shanghai/2/2013 (SH-H7N9) by serial passaging in Madin-Darby canine kidney (MDCK) cells.

**Methods:**

Virus was respectively extracted from cell culture supernatant and cells, and was absolutely quantified by using real-time polymerase chain reaction. Viral RNAs were extracted and subjected to sequencing for identifying mutations. Then, site-specific mutations introduced by viral passaging were selected for further constructing HA7 or NA9 mutant plasmids, which were used to generate recombinant viruses. The interaction between the recombinant HA and receptors, H7N9-pseudotyped viruses and receptors were detected.

**Results:**

Both subtypes displayed high variability in replicative capability and virulence during serial passaging. Analysis of viral genomes revealed multiple amino acid mutations in the hemagglutinin 7 (HA7) (A135T [AH-H7N9], T71I [SH-H7N9], T157I [SH-H7N9], T71I-V223I [SH-H7N9], T71I-T157I-V223I [SH-H7N9], and T71I-T157I-V223I-T40I [SH-H7N9]), and NA9 (N171S [AH-H7N9] and G335S [AH-H7N9]) proteins in various strains of the corresponding subtypes. Notably, quite a few amino acid substitutions indeed collectively strengthened the interactions between H7N9 strains and sialic acid receptors. Moreover, some of the amino acid substitutions identified were highly and specifically cytopathogenic to MDCK cells.

**Conclusions:**

This study demonstrated that AH-H7N9 and SH-H7N9 subtypes can acquire enhanced receptor affinity for sialic receptors through novel amino acid substitutions. Such changes in affinitive interactions are conferred by site-specific mutations of HA7 proteins that affect the virulence and pathology of the virus strain, and/or limited compatibility between the host and the virus strain.

## Introduction

Avian and poultry species, particularly wild ducks, swans, gulls, terns and others, are natural hosts for most avian influenza type A virus (IAV). Hemagglutinin (HA) and neuraminidase (NA) are two surface glycoproteins of IAV, which helps the viral genome enter the host cytoplasm and assists in release of newly formed viral progeny from host cells. In current, 18 different subtypes of HA (H1 to H18) and 11 subtypes of NA (N1-N11) have been identified [1–7]. According to viral pathogenicity in poultry, IAV with different subtypes of HA is classified into highly pathogenic avian influenza (HPAI) and low pathogenicity avian influenza (LPAI). H7N9 virus as a previously undescribed influenza A virus causes 132 human infections with 37 deaths in China in February 2013 [8]. Epidemiology studies confirm that human infection with H7N9 virus mainly originates from virus-infected poultry or a contaminated environment [9–11]. Although the low pathogenic H7N9 virus is not lethal in mammals, but readily obtained the 627 K or 701 N mutation in its basic polymerase 2 (PB2) protein upon replication in mammals, causing it to become highly lethal in mammals [9]. Furthermore, H7N9 virus kept undergoing continuous evolution through reassortment for increasing its genetic diversity [12–14]. Some strains have seemed to involve into highly pathogenicity during recent epidemics in human cases [14–17]. Analysis of the evolutionary relationships of six internal genes of the previous epidemic waves of H7N9 viruses revealed four major clades of PB2 and PB1 genes, three clades of NP and M genes, and two major clades of PA and NS genes [3, 4, 18].

HA protein of H7N9 is a dual-specificity ligand for both human- and avian-type receptors. H7N9 virus strains containing HA mutations in the cleavage site potentially show increased pathogenicity and lethality. For instance, HA Q226L [19–21] and G186V [19, 22–24] mutations were shown to increase the affinitive binding between H7N9 viruses and human-type receptors. Moreover, recent studies showed that N2 with a D151G amino acid substitution [25–27], or N1 with a G147R amino acid substitution [26, 28, 29], had similar properties of enhanced affinitive binding for SA receptors. However, further investigation is required to explore whether the pathology of H7N9 infections could be caused by other amino acid substitutions, resulting from unknown conformational variations in dual-receptor binding capacity of the H7 protein, or additional affinity owing to the N9 protein.

In our study, we developed MDCK cell-infection and sequencing assay, aimed to identity novel amino acid substitutions in HA contribute to virulence enhancement through binding with SA receptors in H7N9 virus strains.

## Methods

### In vitro passaging of H7N9 virus strains

All experiments were strictly performed following the standard procedures of Biological Safety Level 1–3 Laboratory. Two H7N9 virus strains, namely A/Anhui/1/2013 (AH-H7N9) and A/Shanghai/2/2013 (SH-H7N9), were provided by the virus resource database, and were isolated from chicken embryos at the Chinese Center for Disease Control and Prevention (China CDC). Following treatment of chick embryos from embryonic (E) day E9 to E10 with these viruses, chick embryos were incubated at 35 °C for 72 h, and placed at 4 °C overnight. The first transfer generations of AH-H7N9 and SH-H7N9 (P0 strain) were collected from allantoic fluid components in chick embryos. Additionally, simultaneous measurement of infectious virus titer was carried out by 50% tissue culture infective dose (TCID_50_) for each virus strain. Madin-Darby canine kidney (MDCK) cells were selected as the infection model for the generation of each virus strain. Briefly, 8 × 10^5^ MDCK cells were seeded in a 25 cm-flask containing DMEM with 10% FBS, 100 μL/mL penicillin and 100 μL/mL streptomycin (pH 7.2–7.4). MDCK cells were cultured at 37 °C in a humid environment containing 5% CO_2_ until 70% confluence, and washed with TPCK-trypsin (2 μg/mL)-containing DMEM for 3 times. Then, 10 μL of allantoic fluid containing the P0 strain was inoculated and cultured with MDCK cells as described above for 2 h. Afterweards, the medium was discarded, and TPCK-trypsin (2 μg/mL; 5 mL)-containing DMEM was added in cells, which were cultured for another 48 h. Finally, the cytopathic effect (CPE) of the virus was observed under an inverted microscope. Next, the cultures were centrifuged for 10 min and supernatants as well as cells were collected. Absolute quantification was performed for virus in titers in supernatants and cells. Determination of 50% tissue culture infective dose (TCID_50_) and whole-genome sequencing were performed for supernatants and cell viruses, respectively. During long-term serial passaging in the host, viruses from the supernatants were collected, and inoculation was performed as described above, with a constant inoculation amount of 8 × 10^4^ copies. Routinely, cells were assessed and passaged every 48 h. Viruses in the culture supernatants were passaged repeatedly till the sixth generation in MDCK cells, suggesting the generation of mutant viruses (Additional file 1: Fig. 1). Using an identical passaging protocol, serial passaging was also carried out repeatedly in A549 cells till the third generation. The TCID_50_ was measured and calculated by the Reed-Muench method [30], as logTCID_50_ = log (dilution where % of infected cells above 50%) + distance ratio × log (dilution coefficient).

### Absolute quantification of virus titers by real-time RT-PCR

Virus was extracted from a total amount of 100 μL cell culture supernatant with QIAGEN RNeasy Mini Kit. The extracted viral RNA was finally dissolved in 100 μL eluent. Then, a total amount of 1 μL viral RNA was fluorescently quantified by real-time PCR. Rapid and sensitive quantification of 1 μL viral RNA was carried out by continuous fluorescent monitoring of real-time PCR using Multiplex Real Time PCR with Influa H7N9 Detection Kit, whose standard procedures were provided by the National Influenza Center. AgPath-ID™ One-step RT-PCR (Life Technologies, AM1005) was performed with H7 labeled by FAM™ dye on an Applied Biosystems 7500 Fast Dx Real-Time PCR instrument at the conditions of 10 min at 45 °C, 10 min at 95 °C, 40 cycles of 15 s at 95 °C, 45 s at 60 °C. Absolute amounts of viral RNA in were determined by the 7500 software v2.0 according to the HA standard curve.

### Sequence analysis of viral RNAs

Primers for overlapping PCR were designed, and further used for amplification reactions with the Qiagen one step RT-PCR kit. The standard PCR procedure was as follows: 30 min at 50 °C, 15 min at 95 °C, 40 cycles of 30 s at 94 °C, 30 s at 50 °C, 1 min at 72 °C and 10 min at 72 °C, respectively. PCR products were collected, and all gene segments from each virus passage replicated in embryonated eggs were amplified by high fidelity PCR using QIAGEN One Step RT-PCR Kit in the supernatants of homogenized cells and the cell supernatants. The expression vector, pGEM-T Easy Vector (Promega, Madison, USA), was designed for the convenient cloning of the purified PCR-amplified RNAs. Five positive clones for each gene segment from individual virus subtype were randomly selected for sequencing (Invitrogen, Shanghai, China). Assembly and analysis of the sequences was performed with DNASTAR-Lasergene’s SeqMan software and NCBI-Blast.

### Recombination of viral protein mutants

Gene sequences of the HA7 and NA9 proteins isolated from AH-H7N9 or SH-H7N9 were optimized according to host cell characteristics, and sub-cloned into the pCAGGS plasmid (Genewiz). Then, site-specific mutations introduced by viral passaging were selected for further constructing HA7 or NA9 mutant plasmids using the Quickchange Lighting Site-Directed Mutagenesis kit. Briefly, primers containing site-specific mutations were synthesized. The standard PCR procedure was as follows: 2 min at 95 °C, 18 cycles of 20 s at 95 °C, 10 s at 60 °C, 8 min at 68 °C, and 10 min at 68 °C. DH5α competent ™ cells were transfected with these PCR products, by adding 8 μL PCR product into 50 μL DH5α cells, keeping the mixture on ice for 30 min, followed by heat-shock at 42 °C for 90 s and immediate placement on ice for 2 min. Transfected cells were added into 400 μL LB media and cultured in a 37 °C shaker for 45–60 min. Then, 50–100 μL cultured cells were transferred into ampicillin (100 μg/mL)-containing LB plates overnight at 37 °C, and single clones were picked for gene sequencing. Colonies with site-specific mutation accuracy were further inoculated after a 1000-fold dilution onto ampicillin-containing LB plates and cultured overnight in a 37 °C shaker. Plasmid recovery was performed using Qiagen Endo free Plasmid Maxi Kit according to the instructions provided by the manufacturer.

### Recombinant virus

First, plasmids expressing the HA7 or NA9 proteins were transfected into 293 T cells, respectively. Expression levels of the former protein were detected by Western blot, and the activity of the latter protein was determined with NA Activity Kit. Then, the HA7, NA9 and pNL-4.3-Luc-R-E plasmids were co-transfected into 293 T cells, thereby constructing a pseudo-type influenza virus. Briefly, 293 T cells were inoculated in 6-well plates, allowing rapidly spreading viral infection in cultured cells until 70% confluence. The cells were then washed with PBS, and added to a culture medium with low-serum Opti-MEM (Gibco). Virus packaging was performed with Lipofectamine™ 2000 Transfection Reagent Kit (Life Technologies) according to the manufacturer’s instructions. Briefly, 100 μL solution A was added to 500 ng of each pCAGGS-HA7 and pCAGGS-NA9 plasmids, and 1500 ng of pNL-4.3-Luc-R-E, with 3.5 μL of vectors comprising a strong viral promoter/enhancer sequence. Solution A was rotated for 1 s, and kept at room temperature for 2–5 min. Then, solution B was prepared by adding 10 μL effective transfection reagent into 100 μL plasmid containing the enhancer sequence, and rotated for 10 s. Next, solutions A and B were mixed and kept for 5–10 min, added to 600 μL of Opti-MEM medium. The mixture was slowly and carefully added into 6- well plates containing cultured 293 T cells, after discarding the medium. After 6 h of incubation, the supernatant was aspirated, fresh Opti-MEM containing TPCK-trypsin (Sigma) at 2 μg/mL was added. Supernatants containing the H7N9-pseudovirus were collected after 48 h of culture, filtered with a 0.45-μm filter (Millipore) and stored at − 80 °C. Since the pNL-4.3-Luc-R-E plasmid contained the luciferase gene as a marker, the activity of the packaged virus was determined with the Firefly Luciferase Reporter Gene Assay kit. Briefly, all H7N9 pseudoviruses were respectively incubated with MDCK cells cultured in serum free DMEM containing TPCK-trypsin (2 μg/mL) in 96 well plates (10^4^ /well) for 2 h at 37 °C in an environment containing 5% CO_2_. Then, the medium was replaced with 10% FBS supplemented DMEM (2 μg/ml TPCK-trypsin) for another 48 h of culture. Luciferase activity was measured with luciferase assay regents (Promega, Madison, WI), NA activity was determined with a NA-Star Influenza Neuraminidase Inhibitor Resistance Detection Reagent Set (Applied Biosystems) and a Luminescence Counter (Promega) according to the manufacturer’s instructions.

### Western Blot

Cultured cells were washed with PBS twice, lysed with lysis buffer (20 mM Tris, pH 8.0, 0.5% NP-40, 0.25% sodium deoxycholate, 1 mM EDTA, protease inhibitor cocktail), centrifuged at 15,000*g* for 15 min, and supernatants were collected for protein quantification by the Lowry’s method. After diluting the samples to 2 mg/mL, proteins were heat-denatured for 5 min, submitted to 4–10% SDS-PAGE, transferred onto a PVDF membrane, blocked with 1% bovine serum albumin, and incubated with H7 monoclonal antibody (1000; mouse anti-H7N9 hemagglutinin/HA antibody, Sino Biological, Beijing) at 4 °C overnight. The membrane was then incubated with anti-mouse IR dye 800-labeled IgG secondary antibody (Li-Cor, Lincoln, NE), measured at a recommended wavelength, and analyzed by the Odyssey software.

### Interaction detection between the recombinant HA and receptors

HA quantification was first performed following the instructions provided by the manufacturer. Recombinant HAs were coated onto a polystyrene plate at 4 °C overnight. The AH-H7N9 hemagglutinin/HA protein (Sino Biological, Beijing) was used as the standard and serially diluted. The plate was then blocked with 5% bovine serum albumin (BSA) (Sigma) at 37 °C for 2 h. After washing, mouse anti-HA mAbs (1:1000) were added, and the plate was incubated at 37 °C for another hour. The plate was incubated with HRP-labeled secondary anti-mouse IgG (1:5000) (Sigma) at 37 °C for another hour following washing. OPD/H_2_O_2_ was then added; the chromogenic reaction was terminated by addition of H_2_SO_4_. The amounts of the HA protein were measured at 450/650 nm, using a standard curve.

A solid-phase binding assay with competing glycopolymers was performed for determining receptor affinity as described previously. Serially diluted HA proteins (threefold) from 200 μg/mL (3.4 μM) were allowed to incubate overnight at 4 °C in 96-well plates. BSA (5%) was used to block the plates for 2 h at 37 °C. Then, [3′-Sialyllactose-PAA-biotin, 3′SL] (GlycoTech, 01-038) or [6′-Sialyllactose-PAA-biotin, 6′SL] (GlycoTech, 01-039) was added to the wells at 200 ng/mL for 1 h at 37 °C, respectively. Horseradish peroxidase (HRP)-conjugated avidin (1:5000; Sigma) was added to wells for visual detection of the proteins by a chromogenic reaction. The extent and accuracy of binding of the recombinant HA by a given polysaccharide receptor was determined by absorbance at 450/650 nm. Data were analyzed by the GraphPad Prism 5.0 software.

### Interaction detection between H7N9-pseudotyped viruses and receptors

Interaction detection between recombinant viruses and receptors was performed with the HIV-1 p24 Antigen ELISA kit (Keybiotech, Beijing), by assessing receptor-labeled 3′SL and 6′SL, according to the manufacturer’s instructions. Briefly, p24-antigen containing- and standard samples were added to p24 McAb-coated 96-well plates and incubated at 37 °C for 60 min. Following incubation, the plates were washed, and biotin-labeled p24 polyclonal antibody and HRP-labeled SA were added for further studies. After 5× washing, H_2_O_2_/DAB was added, and the reaction was terminated 15 min later. Virus quantification was carried out by detecting OD at 450/650 nm. Then, p24 protein levels were adjusted to 200 ng/mL after dilution of the virus, which was coated to 96-well plates as described above. The 3′SL and 6′SL were twofold serially diluted from 200 ng/mL, allowing the binding detection of HRP-SA by DAB and OD measurement, as described above for the solid-phase binding assay between HA and glycol- polymers.

### Statistical analysis

The Student’s t-test was utilized to determine the statistical significance of differences between experimental groups. *p* < 0.05 was considered as statistical significant.

## Results

### Total amounts and virulence strengths of viruses in MDCK-infected cells

**Fig. 1:**
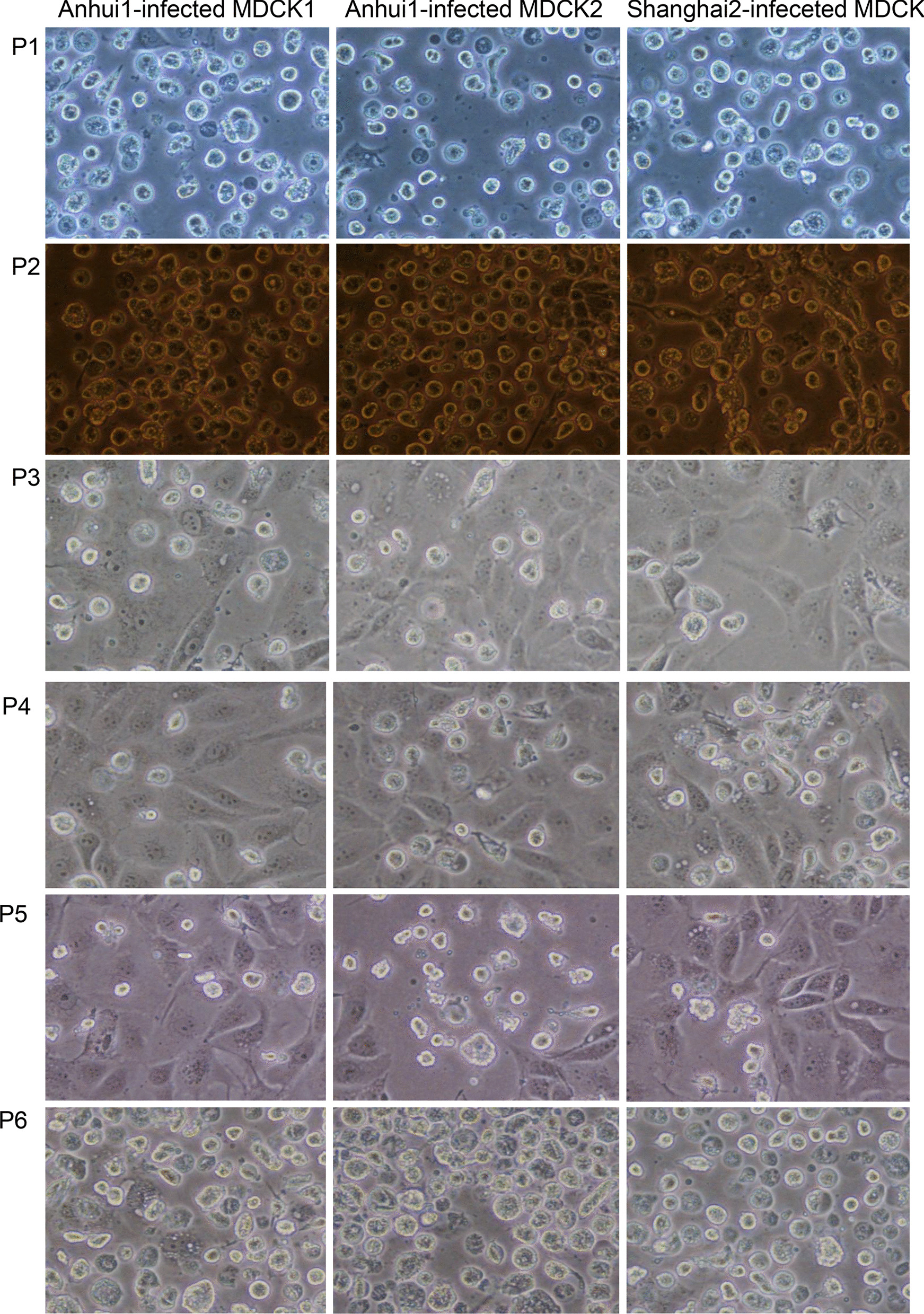
Pathological changes in MDCK cells infected by various virus generations for 48 h

**Fig. 2:**
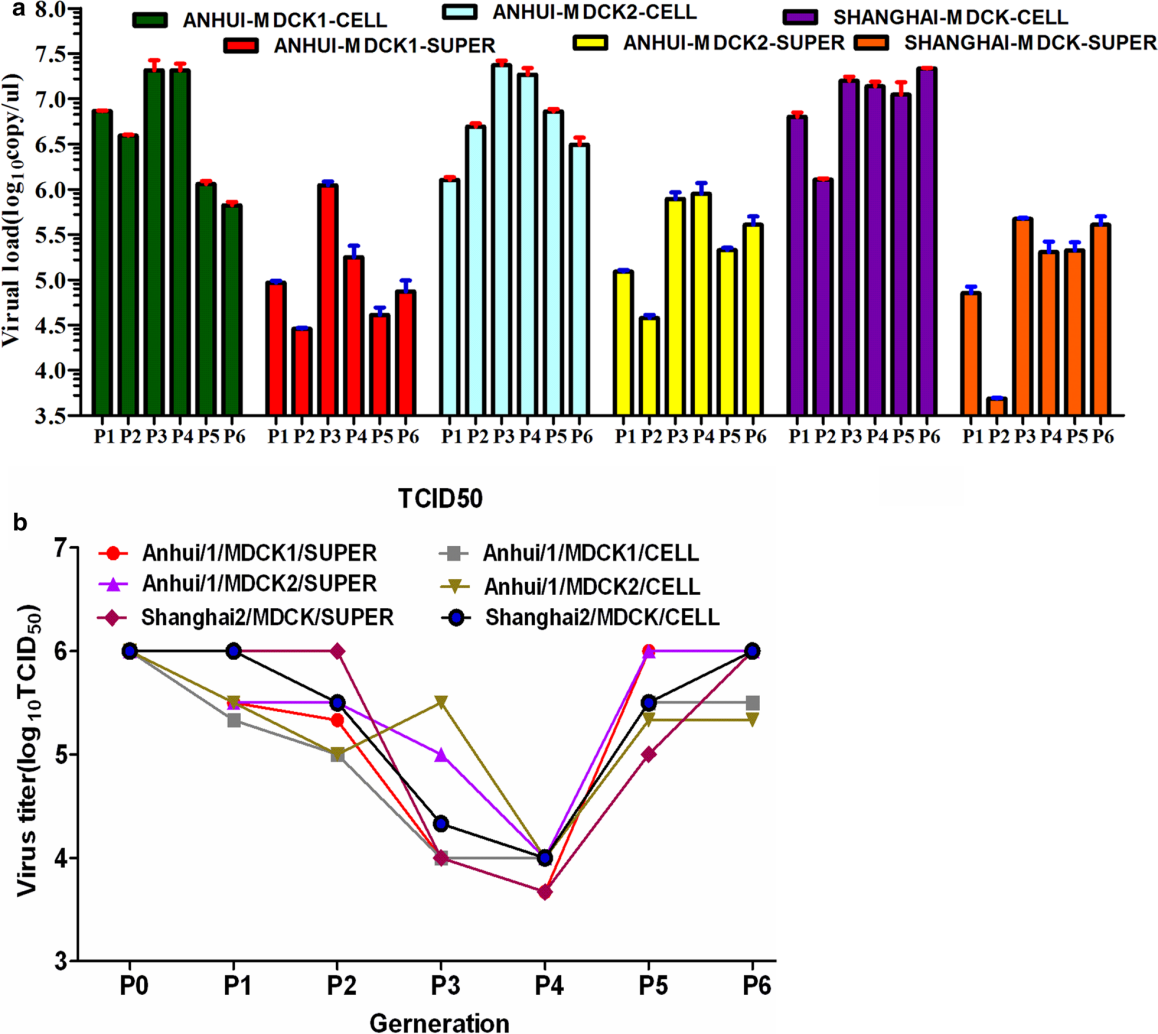
Virus quantification. (**a**) Absolute RNA amounts in viruses from supernatants or cells, quantified by quantitative PCR; (**b**) The virulence of viruses (from supernatants or cells) in MDCK cells was quantified by measuring TCID50

First, we assessed the morphological lesions associated with H7N9 virus infection. MDCK cells were rounded and shrunk after 48-h of infection with first- or second-generation H7N9 viruses; this effect was even more prominent after infection with fifth- and sixth-generation viruses (Fig. 1). However, pathology in MDCK cells did not appear when infected with third- or fourth-generation viruses (Fig. 1). Besides, primer pairs, designed against segment specific RNAs for each generation in different H7N9 subtypes (Additional file 1: Fig. 1), were used to quantitatively assess copy numbers by *in-vitro* Real-Time PCR (Fig. 2a). Quantification of absolute amounts of viral RNAs indicated a significant increase in supernatants with a significant decrease in cells infected by fifth- and sixth-generations of A/Anhui/1/2013 (AH-H7N9), respectively. These results suggested that A/Shanghai/2/2013 (SH-H7N9) displayed apparently higher replicative ability compared with AH-H7N9 in MDCK cells. For instance, total amounts of viral RNAs in second-generation of AH-H7N9 were significantly reduced compared with those in fifth- or sixth-generation of SH-H7N9 in MDCK cells (Fig. 2a). Interestingly, this effect was not replicated in the other cells tested, namely, A549 cells, indicating host specificity of H7N9 viruses and various adaptive responses (Additional file 2: Fig. 2).

To determine the TCID50 of each generation for various viral subtypes, endpoint dilution assay was used to estimate the amounts of virus required to kill 50% of infected MDCK cells or induce cytopathic effects in 50% of inoculated MDCK cells. Cultured MDCK cells were first added with serial dilutions of respective viruses. Quantification of TCID_50_ of viruses using the Reed-Muench method showed that viruses isolated from either cells or supernatants displayed similar TCID50 for each virus dilution for all subtypes (Fig. 2b). In addition, TCID_50_ of the AH-H7N9 strain decreased in the first- and second-generations, respectively, and reached the minimum in the third- and fourth-generations, indicating that the virus required time to adapt to the changing environment. Moreover, TCID_50_ measurement also revealed that the virulence was restored in the fifth-generation and maintained the in sixth-generation (Fig. 2b). TCID50 analysis of the SH-H7N9 subtype also revealed a similar estimation of long-term trend except for relatively high TCID_50_ for the first- and second-generations of SH-H7N9, suggesting that SH-H7N9 was more adaptive to the new environment in comparison with AH-H7N9 (Fig. 2b).

### Full gene sequences of the H7N9 virus in the P0-P6 passages

Primers for overlapping PCR were designed, and used for sequencing analysis of each gene segment from individual recombinant viruses of various subtypes in the sixth generation. We identified point mutations either causing amino acid substitution or not (Table 1). Sequence analysis revealed that the Anhui/1-H7N9 and Shanghai/2-H7N9 strains showed significant differences involving in mutations of the gene, even when cultured in the same conditions. Some site-specific mutations were introduced into the genes of 8 segment proteins of the Anhui/1-H7N9 and Shanghai/2-H7N9 strains. Eleven meaningful bases mutations were discovered in HA, NA, PA, PB1, PB2, and NP completed genes within AH-H7N9 strains. While Seventeen sense bases mutations were found in HA, NA, PA, PB1, PB2, NP and NS completed gene within SH-H7N9 strains. These site-specific mutations were introduced into the genes of HA7, NA9, PA, PB1, PB2, NP and NS proteins of AH-H7N9 and/or SH-H7N9. Eleven meaningful amino acid mutations were HA (A135T), NA (N171S, G335S and G415K), PA (A343T, A347T and E349T), PB1 (D76N and V451E), PB2 (D87G), and NP (A373T) respectively within Anhui/1-H7N9 serial passaging strains. While Seventeen sense amino acids mutations were respectively HA (T40I, A107T, T157I, G218E, V223I, and A225K), NA (N72I), PA (M86V and Y161A), PB1 (S515F and P596S), PB2 (I185S), NP (A284T and M352I) and NS (D101N and E208K) completed proteins within Shanghai02-H7N9 strains. Here all amino acid sits of HAs and NAs were numbered throughout by classical H3N2 strain numbering. These amino acids mutation involved in Anhui/1 and Shanghai/2 H7N9 in the serial passaging strains were analyzed in the Flusurver website (http://flusurver.bii.a-star.edu.sg). Compared with the reference A/Shanghai/2/2013 (H7N9) strain, mutations occurred in the Anhui/1 virus strain at the sites of the M26I, S171N and G335N of NA proteins, A135T in HA, and A373T of NP predicted biological significance. Meanwhile, mutations occurred in Shanghai/02 at the following sites of the T40I, A107T, A135T, T157I, G218E, V223I, and A225K in HA genes, M86V, Y161A in PA, A284T in NP, N72I in NA, and D101N in NS primarily predicted the sites of interest. According to the above the results, the HA and NA gene mutations involved in receptor-binding properties of the virus mainly contributed to virulence alteration during serial passaging. The 3D structures of interactions between the HA protein with amino acid substitution and receptors were analyzed by Flusurver (https://flusurver.bii.a-star.edu.sg/INTERACTIONS/) (Fig. 5). Crystal structure of NA with amino acid substitution from SH-H7N9 was predicted (Fig. 6).

**Table 1:** Amino acid substitutions or simple point mutations without accumulation of amino acid substitutions by using analysis of whole-genome sequencing of sixth-generation of viruses in each virus strain from both supernatants and cells

H7N9 type	Gene	No. of mutation	Base mutation	AA mutation	Change during passaging	Mutation coverage
Anhui/1	HA	1	C96T	NS	C in P0,occurred in P1-6, T>C in P3	In MDCK1 and MDCK2, both MIX
		2	G421A	NS	G>A only in p1-5 A, recovered in P6	Only in MDCK2, MIX
		3	G427A	A143T/135*	G in P0,A occurred in P1,A>G in P3,G>A in all P4-6 virus	Complete mutation in MDCK1, MIX in MDCK2
		4	C1554T	NS	C in P0, T occurred in P1-2,T>C in P3-6	In both MDCK1 and MDCK2; MIX
		1	G503A	N168S/171^#^	G in P0-1, A occurred in P2-3, A maintained in P4-6	In both MDCK1 and MDCK2;MIX
	NA	2	G996A	G331S/335^#^	G in P0-1, A occurred in P2-3 and maintained in P4-6	In both MDCK1 and MDCK2;MIX
		3	G1236A	G412K/415^#^	G in P0-1, A occurred in P2-3 and maintained in P4-6	In both MDCK1 and MDCK2;MIX
		1	G1027A	A343T	G in P0-3, A occurred in P4, A maintained in P5-6	In both MDCK1 and MDCK2; MIX
	PA	2	G1039A	A347T	G in P0-3, A occurred in P4, A maintained in P5-6	In both MDCK1 and MDCK2; MIX
		3	A1046G	E349G	G in P0-3, A occurred in P4, A maintained in P5-6	In both MDCK1 and MDCK2; MIX
		4	C135T	NS	C in P0, T occurred in P1, T maintained in P4-6	In both MDCK1 and MDCK2; MIX
		5	C720T	NS	C in P0, T occurred in P1, T maintained in P4-6	In both MDCK1 and MDCK2; MIX
		6	C1557T	NS	C in P0, T occurred in P1, T maintained in P4-6	In both MDCK1 and MDCK2; MIX
	PB1	1	G226A	D76N	G in P0-3, A occurred in P4, A maintained in P5-6	In only MDCK1, Mix
		2	T1356A	V451E	T in P0-3, A occurred in P4, A maintained in P5-6	In only MDCK1 Mix
	PB2	1	A260G	D87G	A in P0-3, G occurred in P4, G maintained in P5-6	In only MDCK1 Mix
		2	C990A	NS	C in P0, A occurred in P1-3, C maintained in P4-6	In both MDCK1 and MDCK2; MIX
	NP	1	G1117A	A373T	G in P0-4, A occurred in P5, A maintained in P6	In both MDCK1 and MDCK2; MIX
		2	G1071A	NS	G in P0, A occurred in P1, A maintained in P2-6	In both MDCK1 and MDCK2; MIX
	M	1	C900A	NS	C in P0-5, A occurred in P6	In only MDCK2 Mix
Shanghai/2	HA	1	C143T	T48I/40*	C in P0-2,T occurred in P3, T maintained in P4-6	Mix
		2	C236T	T79I/71*	C in P0-2,T occurred in P3, T maintained in P4-6	Mix
		3	G343A	A117T/107*	G in P0-2,A occurred in P3, A maintained in P4-6	Mix
		4	C494T	T165I/157*	C in P0-2,T occurred in P3, T maintained in P4-6	Mix
		5	G680A	G227E/218*	G in P0-2,A occurred in P3, A maintained in P4-6	Mix
		6	G694A	V237I/223*	G in P0, A occurred in P1,A maintained in P2-4, recovered in P5-6	Mix
		7	G1091A	A369K/225*	G in P0-2,A occurred in P3, A maintained in P4-6	Mix
		8	T618C	NS	T in P0-3:, T>C in P4:, P5:C>T,P6:T>C	Mix
		9	G1011A	NS	P0-3:A>G, P4:G>=A,P5-6 converted to P0(G)	Mix
	NA	1	C207T	N73I/72	C in P0, T occurred in P1,T maintained in P2-4, recovered in P5-6	Mix
	PA	1	A256G	M86V	A in P0, G occurred in P1,G maintained in P2-3, recovered in P5-6	Mix
		2	A484G	Y161A	A in P0,G occurred in P1,G maintained in P2-5,G in P6	Complete mutation in P6
	PB1	1	C1544T	S515F	C in P0-2,T occurred in P3,G maintained in P4-6	Mix
		2	C1786T	P596S	C in P0-2,T occurred in P3,G maintained in P4-6	Mix
		3	C1143T	NS	P0-3:C, P4:C>T,P5-6:T>C	
		4	C1488T	NS	P0-3:C, P4:C>T,P5-6:T>C	
		5	G2061A	NS	P0-2:G, P3:G>A,P4:A>G,P5-6:G>A	
	PB2	1	T554G	I185S	T in P0-P3,G occurred in P4,G maintained in P5-6,G in P6	Complete mutation in P6
		2	C393T	NS	P0-3:C>T,P4-6 return to P0(C)	
		3	C717T	NS	P0-3:C>T,P4-6 return to P0(C)	
		4	G573A	NS	P0-4:G>A,P5-6 return to P0(G)	
	NP	1	G850A	A284T	G in P0, A occurred in P1,A maintained in P2-4, A in P5-6	Complete mutation in P5-6
		2	G1056A	M352I	G in P0, A occurred in P1,A maintained in P2-4, A in P5-6	Complete mutation in P5-6
		3	G987A	NS	P0-6:G>A, G=A in P3,P6 weaken	Mix
		4	C1020A	NS	P0-4:C>A, P5-6 converted into P0(C)	Mix
	NS	1	G301A	D101N	G in P0-P2, A occurred in P3-4, recovered in P5-6	Mix
		2	G622A	E208K	G in P0-P2, A occurred in P3-4, recovered in P5-6	Mix
		3	G651A	NS	P0-3:G,P4-6:G>A	Mix

*H7/H3 numbering; #N9/N2 numbering; NS: Only base mutations, but not amino acid mutations

### Interactions of recombinant HAs with receptors

**Fig. 3:**
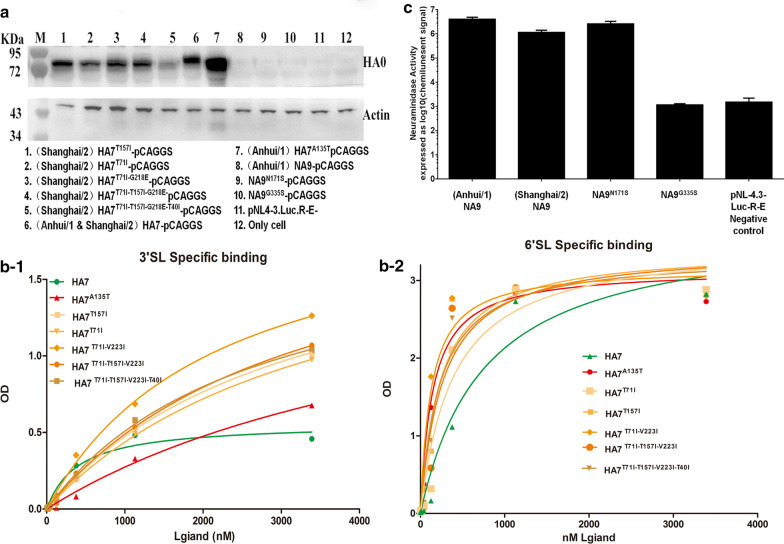
Interaction assessment between HA7 protein and its receptors. (**a**) Expression levels of the HA7 protein in 293 T cells, detected by Western blot; (**b**) Quantitative detection of *HA* binding to 3′SL- (**b-1**) or 6′SL-labeled receptors (**b-2**) by measuring KD values. (**c**) Quantification of NA activity was determined by measuring optical density generated in the chromogenic reaction

Next, these gene sequences were optimized and subcloned into the pCAGGS plasmid according to the features of MDCK cells. Firstly, we tested the expression levels of a variety of recombinant HA7 plasmids in 293 T cells. The recombinant variants of (AH-H7N9) HA7^A135T^-pCAGGS exhibited overtly higher expression levels compared with those of other variants. Meanwhile, (AH&SH-H7N9) HA7^A135T^-pCAGGS and (SH-H7N9) HA7^T157I^-pCAGGS displayed relatively higher expression levels compared with average expression levels of (SH-H7N9) HA7^T71I^-pCAGGS, (SH-H7N9) HA7^T71I-V223I^-pCAGGS and (SH-H7N9) HA7^T71I-T157I-V223I^-pCAGGS, as well as (SH-H7N9) HA7^T71I-T157I-V223I-T40I^-pCAGGS, which showed lowest expression levels. Moreover, the NA variants, pNL-4.3-Luc-R-E vector and blank load transfections showed no detectable HA protein expression (Fig. 3a).

To determine whether the site-specific HA7 mutations identified affected HA affinity for glycopolymer receptors, interactions between recombinant viruses and receptors were determined by counting labeled “avian-type” receptor-3′SL, and the major analog of the human receptor-6′SL. Based on KD values, the wild-type HA7 protein in both H7N9 subtypes exhibited dual receptor-binding properties since they had the same substitution, with similar affinities (KD for 3′SL_Anhui/1 & Shanghai/2 HA7_ = 0.635 ± 0.188 μM to the “avian-type” receptor 3′SL and KD for 6′SL_Anhui/1 & Shanghai/2 HA7_ = 0.753 ± 0.197 μM to the “human-type” receptor 6′SL). Surprisingly, all HA mutations retained the ability to bind the “avian-type” receptor analog, of which HA^A135T^ had the lowest affinity (KD for 3′SL_HA7_^A135T^ = 5.553 ± 1.232 μM), taking into account average levels of affinitive interactions of other variants to 3′SL (Fig. 3b-1; Table 2). Moreover, affinity test also showed highest affinity for the HA7^T71I-V223I^ and HA7^A135T^variant (KD for 6′SL _HA7_^T71I-V223I^ = 0.127 ± 0.028 μM and HA7^A135T^ = 0.147 ± 0.031 μM to the major analog of the human receptor 6′SL), as well as relatively low affinity for HA7^T71I^ (KD for 6′SL HA7^T71I-V223I^ = 0.388 ± 0.102 μM), in comparison with average affinity for other variants (Fig. 3b-2; Table 2). Furthermore, we analyzed the NA activity of packaged viruses by measuring the expression levels of luciferase reporter gene, after transfection of pNL-4.3-Luc-R-E plasmids containing the sequences of NA9 variants into 293 T cells. We found similarly higher levels of NA activity of the NA9^N171S^ variant and wild type proteins in both subtypes in comparison with those of NA9^G335^ or the negative control (Fig. 3c).

**Table 2:** Mean KD values through One site-Specific binding analysis for interactions between HA antigen protein and a serial dilution of either 3′SL- or 6′SL-labeled receptors in each HA expression protein

HAs	KD FOR 3′SL (μM)	KD FOR 6′SL (μM)
Anhui/1 & Shanghai/2 HA7	0.635 ± 0.188	0.753 ± 0.197
HA7 ^A135T^	5.553 ± 1.232	0.147 ± 0.031
HA7 ^T71I^	3.627 ± 0.518	0.388 ± 0.102
HA7 ^T157I^	3.176 ± 0.921	0.231 ± 0.051
HA7 ^T71I-V223I^	2.189 ± 0.518	0.127 ± 0.028
HA7 ^T71I-T157I-V223I^	2.189 ± 0.204	0.268 ± 0.081
HA7 ^T71I-T157I-V223I-T40I^	3.317 ± 0.300	0.231 ± 0.051

### Virus infectivity and pathogenesis

**Fig. 4:**
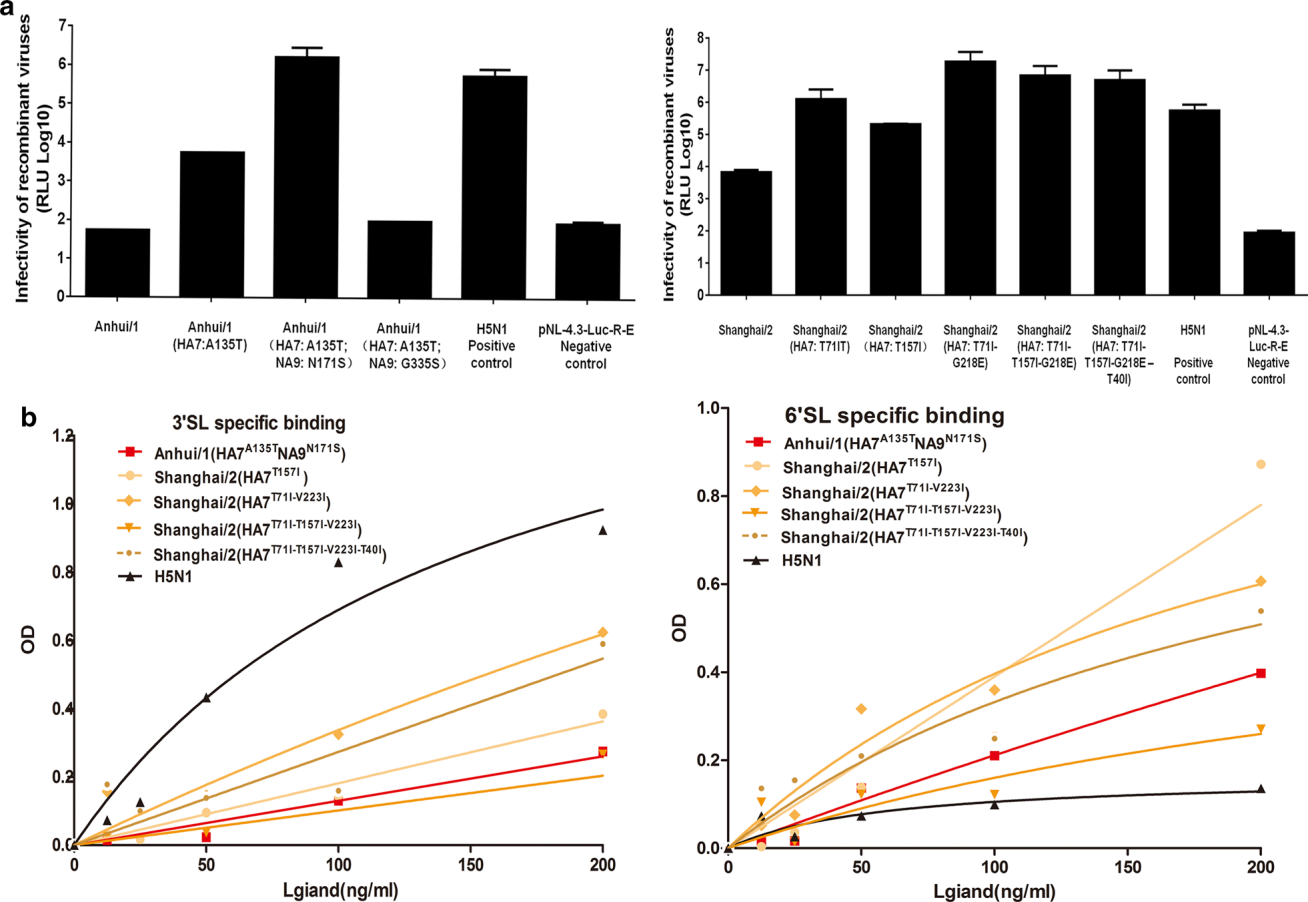
Infectivity of recombinant H7N9 virus. (**a**) Quantification of viral infectivity by measuring luciferase activity; (**b**) Quantitative detection of the interaction between recombinant HA and 3′SL- or 6′SL-labeled receptors by measuring KD values

We performed luciferase assays in cultured MDCK cells infected with H7N9 variants. Virus infectivity in MDCK cells was expressed as the logarithm conversion of relative luciferase units (Log_10_RLUs). The results showed that mean values in the AH-H7N9 (HA7: A135T; NA9: N171S), SH-H7N9 (HA7: T71I-V223I), SH-H7N9 (HA7: T71I-T157I-V223I), SH-H7N9 (HA7: T71I-T157I-V223I-T40I), and SH-H7N9 (HA7: T71I) virus strains ranged between 6 and 8. Among them, SH-H7N9 (HA7: T71I-V223I) was the only virus strain with a mean value of Log_10_RLU higher than 7. Moreover, mean values in the AH-H7N9 (HA7: A135T), SH-H7N9 (HA7: T157I), SH-H7N9 wild type, and H5N1 wild type virus strains ranged between 3 and 6. The remaining variants showed no significant differences compared with the negative control (Fig. 4a).

To further determine the infectivity of H7N9 recombinant strains with amino acid substitution(s), a solid-phase binding assay with competing glycopolymers was performed for measuring receptor affinity (Fig. 4b). We performed the solid-phase binding assay between receptors and pseudoviruses of different variants. In comparison with the strongest interaction between H5N1 and 3′SL, SH-H7N9 (HA7: T71I-V223I) and SH-H7N9 (HA7: T71I-T157I-V223I-T40I) also strongly enhanced “avian-type” receptor (3′SL)-mediated interactions in a dose-dependent manner. Moreover, SH-H7N9 (HA7: T157I) and AH-H7N9 (HA7: A135T; NA9: N171S) interaction was somewhat reduced compared to those two mentioned above, in particular for the AH-H7N9 (HA7: A135T; NA9: N171S) strain. Meanwhile, SH-H7N9 (HA7: T71I-T157I-V223I) showed lowest affinity among all other strains.

Furthermore, we also tested the interaction between the major analog of the human receptor (6′SL) and the various H7N9 recombinant strains. In striking contrast, SH-H7N9 (HA7: T157I) became the strain with apparently higher affinitive binding associated with interaction between HA7 and 6′SL, in particular for higher concentrations (> 100 nM) of the ligands. SH-H7N9 (HA7: T71I-V223I) and SH-H7N9 (HA7: T71I-T157I-V223I-T40I) also could be effectively and stably bound. Still, AH-H7N9 (HA7: A135T; NA9: N171S), SH-H7N9 (HA7: T71I- T157I-V223I) and H5N1 showed apparently reduced affinity to 6′SL, in particular with the lowest affinity for H5N1, compared with other recombinant strains.

## Discussion

In this study, we developed an MDCK cell-infection assay that allowed the screening of unknown amino acid substitutions that potentially have functional alterations, especially in affinitive binding with SA receptors during serial passaging of both AH-H7N9 and SH-H7N9 subtypes. Sequencing confirmed that we indeed generated several new mutations (Table 1), either in the HA7 or NA9 protein. Some of these new mutations surprisingly exhibited a significant increase in binding affinity with SA receptors, especially the human type, indicating there was a conformational change in essential domains of the protein itself or the protein-receptor complex.

During serial passaging, the Anhui/1-H7N9 and Shanghai/2-H7N9 strains isolated from sputum of patients with H7N9 virus infection were first cultured in egg embryos equivalent to the second-generation embryonic virus strains (E2), named P0. Then, they were transfected into MDCK (Madin-Darby canine kidney) and A549 (human pulmonary carcinoma) cells, respectively. Indeed, Anhui/1-H7N9 and Shanghai/2-H7N9 strains are transmitted by traces, from poultry to humans (patients infected with H7N9 virus), to egg embryos (similar to poultry), then returned to animals (dogs) and humans (A549), resulting in adaptive gene mutations frequently occurring during growth in diverse species recycled between avians, poultry and humans. Interestingly, analysis of host adaptation and resistance mutations revealed that even different H7N9 subtypes had markedly different surviving curves during serial passaging, when infecting MDCK cells, but not A549 cells. it is well known that the five rounds of H7N9 outbreak occurr from 2013 to 2017 (Additional file 3: Fig. 3). The fifth round of H7N9 outbreak in 2017 occurred with a large number of cases, although a series of prevention and control measures in poultry trading have been implemented. Therefore, the possibility of adaptive mutation of carriers infected with H7N9 virus but not developing the disease cannot be ruled out, according to our previous study (data no shown). Furthermore, individuals H7N9 subtypes also displayed significant variations when cells from different human organs were infected, not to mention cells from different species. Nevertheless, genetic lineages of different avian and poultry species, especially the wild ones, provided a number of genes from avian influenza type A viruses including H7N9 to co-circulate. Until now, there is no significant consistency in temporal and/or spatial correlations, due to the fact that, at least partially, wild birds prefer going wild, from time to time. Therefore, in addition to globally monitoring potential candidates of migratory birds and speeding up whole genome sequencing for more avian and poultry species, structural and functional analyses of amino acid variations of potentially high pathogenic H7N9 strains, from one amino acid to another, is indeed required as a high priority.

Analysis of the complete gene showed there were two amino acid difference at NA (M26I) and PB2 (I292V) sites between Anhui/1-H7N9 (p0) and Shanghai/2-H7N9 (p0) strains. P1-P6 gene Flusurver website analysis showed that four of eleven meaningful amino acid mutations were of interest level (1–3), including HA (A149T, H3 numbering), NA (N172S, G333S N2 numbering) and NP (373 T) within Anhui/1-H7N9 serial passaging strains. HA (A149T) may create a potential glycosylation site; NP (NP373T) is known to cause antigenic shifts or mild drug resistance. While a preliminary analysis of seventeen sense mutations revealed twelve site-related functions involved in HA (T47I, T79I, A117T, T171I, G232E, V237I and A369K), NA (N73I), PA (M86V and Y161A), NP (A284T) and NS1 (D101N) within Shanghai02-H7N9 strains. According to the above results, HA and NA gene mutations involved in receptor-binding properties of the virus mainly contribute to virulence alteration during serial passaging.

**Fig. 5:**
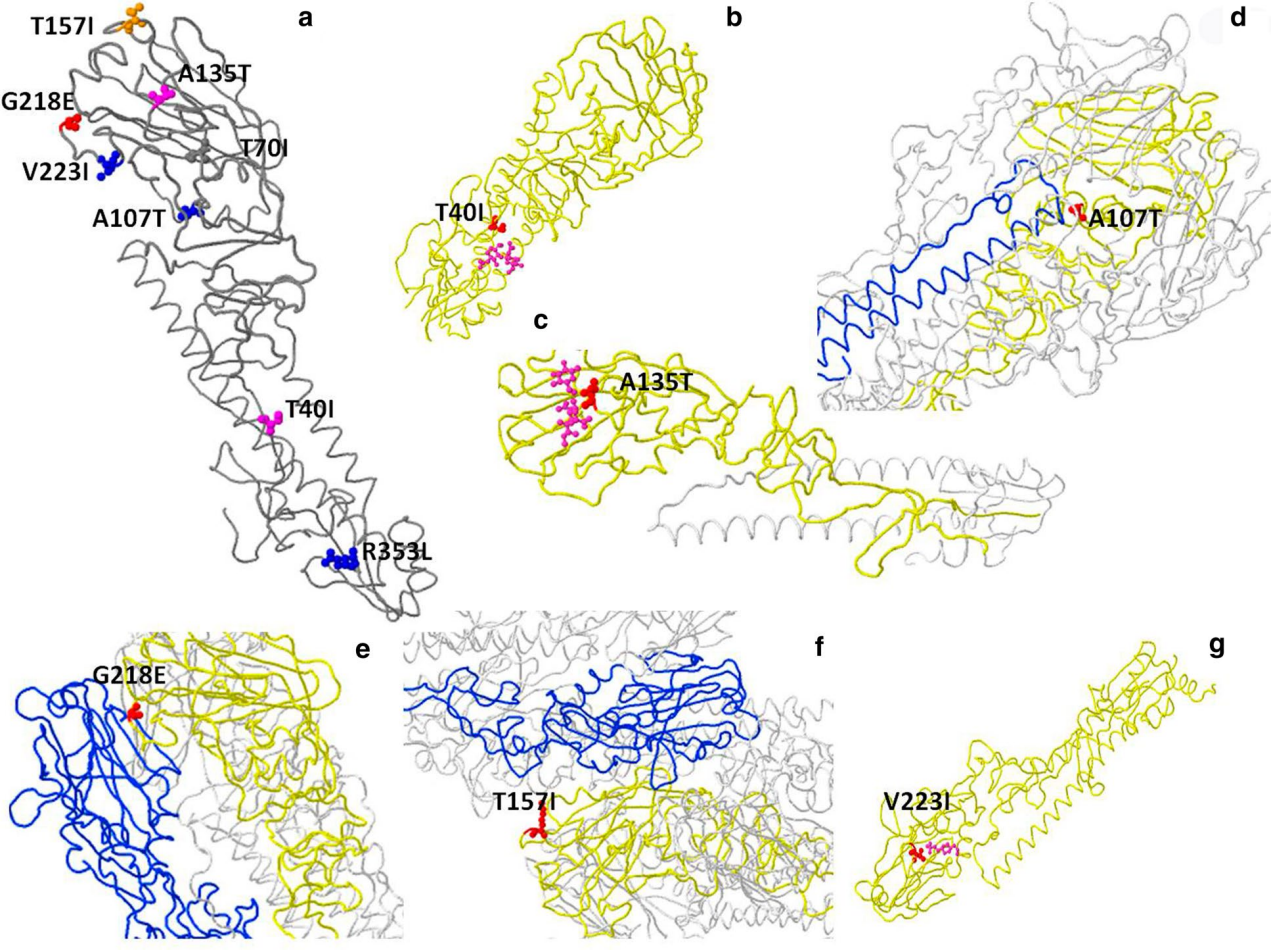
3D structures of interactions between the HA protein and receptors analyzed by Flusurver (https://flusurver.bii.a-star.edu.sg/INTERACTIONS/) (**a**) 3D structures of HA7 color-marked mutation sites and receptors; (**b**) Shanghai/2 HA mutation Thr40Ile and ligand NAG (pink atoms); (**c**) Anhui/1 HA mutation Ala135Thr and ligand GLC (pink atoms), host cell receptor SIA (pink atoms). (**d**) Shanghai/2 HA mutation A107T on viral chain A (yellow backbone), within 5 A from oligomeric subunit chain F (blue backbone); (**e**) Shanghai/2 HA mutation G218E on viral chain A (yellow backbone), within 5 A from oligomeric subunit chain C (blue backbone); (**f**) Shanghai/2 HA mutation T157I on viral chain A (yellow backbone), within 5 A from ligand EPE (pink atoms); (**g**) Shanghai/2 HA mutation V218I on viral chain A (yellow backbone), within 5 A from ligand EPE (pink atoms)

Specifically, it is evolutionarily essential to analyze the mutational pattern of HA, since it acts as the major viral glycoprotein modulating adsorption as well as penetration in the host membrane by binding the 3′SL or 6′SL receptor through specific receptor binding sites known as secondary elements, including helix (190 aa [amino acid]) and loops (130/150/220 aa) based on H3 numbering. For instance, S128N was detected within the loop (130 aa), which is proximal to the receptor surface (distance ≈ 20 Å) [31]. Unexpectedly, A143V and A143T, which are outside the receptor binding site, were also reported to play critical roles, as additional antigenic determinants, in adjusting receptor binding avidity, and simultaneously altering antigenicity in AH-H7N9 [32]. Furthermore, several mutations including L226Q were later found on the internal branch ancestral to the HP cluster within the YRD-2b clade of HA [31]. Both wild type AH-H7N9 and SH-H7N9 retained dual receptor binding ability due to keeping the Q226L type mutation. Accordingly, quite a few mutations of the HA 7 protein found in this study, i.e., A135T (AH-H7N9), SH-H7N9 (G218E, T40I, T157I, A107T and V223R) mutations, were consistent with previous structural biological studies and/or related researches. A previous study showed that the four hydrophobic residues Ala138, Val186, Pro221 and Leu226 have a higher ability to bind the human receptor than the four hydrophilic counterparts Ser138, Gly186, Thr221 and Gln226, in H7N9 HA protein^4^. Interestingly, some mutations of the HA 7 protein found above, i.e., T40I, T79I, T157I and V223I, changed into a hydrophobic residue (isoleucine), indicating that they acquire a higher ability to bind the human receptor. The HA T48I mutation removes a potential N-glycosylation site at position 46, which may also affect antigenic and other properties of this strain. In detail, the motif at positions 46–48 changed from NAT (glyco) to NAI (no glyco). On the contrary, HA A143T mutation, which had higher affinity to 6′SL, created a new potential N-glycosylation site at position 141 which may also affect antigenic and other properties of this strain. In detail, the motif at positions 141–143 changed from NGA (no glyco) to NGT (glyco). As observed in resolved structures of proteins from related strains, the HA position equivalent to this mutation is involved in binding of small ligand(s), antibody recognition sites, or viral oligomerization interfaces (Fig. 5b and c). HA A107T (SH-H7N9) had already occurred (0.15% of all samples with HA sequence) in a strain (A/environment/Korea/MA-410/2016) in 2016. The HA position is involved in viral oligomerization interfaces and a T-cell epitope presented by MHC molecules- antibody recognition sites (Fig. 5c) [18]. Mutations at the position equivalent to HA T157I, HA G218E or HA V223R (SH-H7N9) have been reported for their associations with virulence, antigenic drift and escape mutation. As observed in resolved structures of proteins from related strains, the HA positions equivalent to two mutations are involved in viral oligomerization interfaces, antibody recognition sites and binding of small ligand(s) (Fig. 5e, f and g). HA R364K and HA T70I mutations have been reported many times previously. However, further studies assessing these from structural and functional perspectives are necessary in the future.

**Fig. 6:**
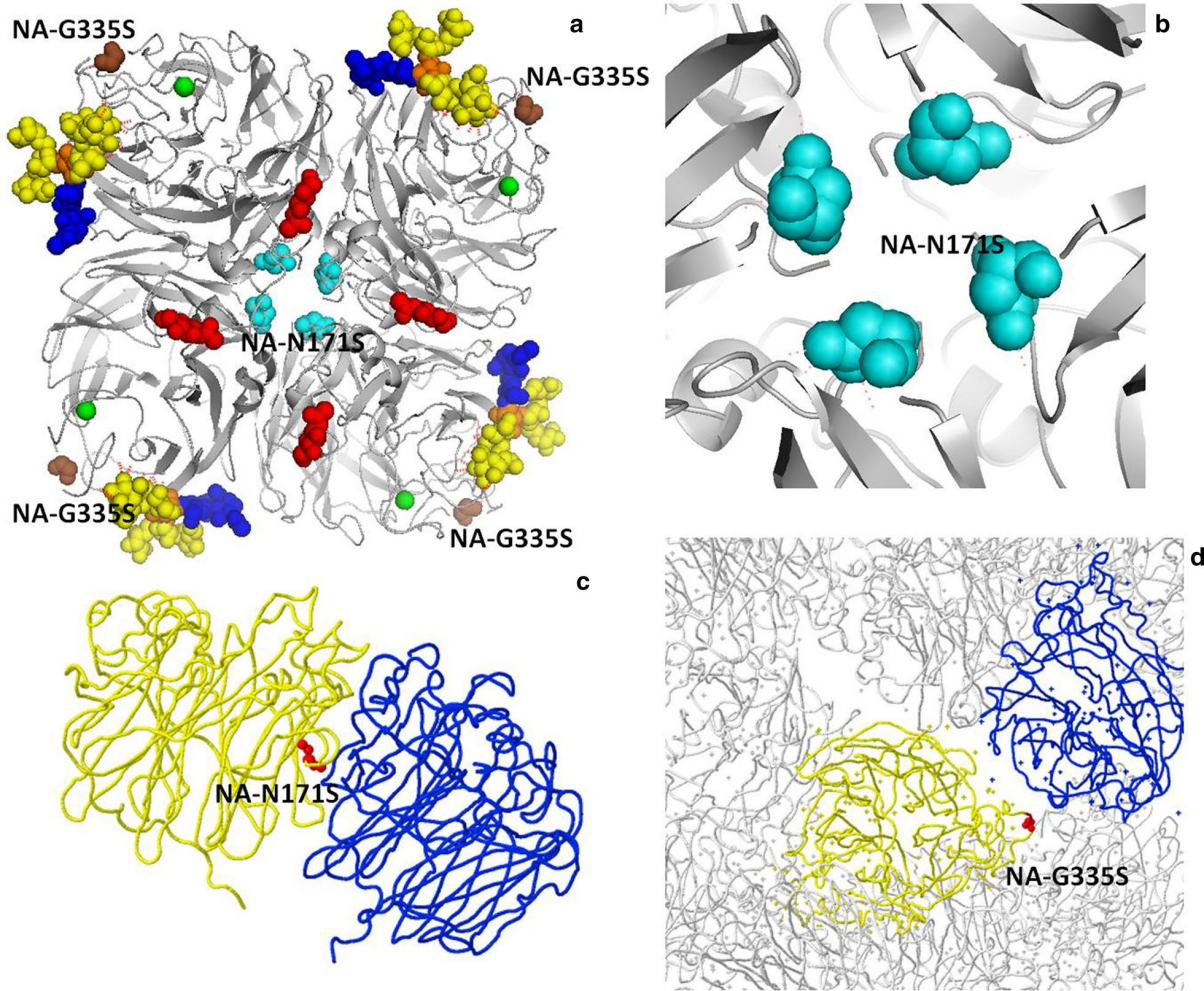
Crystal structure(ID:5L14) of neuraminidase from A/Shanghai/2/2013 (H7N9) influenza virus (http://www.ebi.ac.uk/pdbe/entry/pdb/5l14/experiment). (**a**) 3D structures of NA9 color-marked mutation sites and receptors; (**b**) Anhui/1 NA mutation NA-N171S enlargement; (**c**) The NA-N171S mutation position (red atoms) on viral chain A (yellow backbone), within 5 A from oligomeric subunit chain B (blue backbone). (**d**) The NA- G335S mutation position (red atoms) on viral chain B (yellow backbone), within 5 A from oligomeric subunit chain E (blue backbone)

On the other hand, the indispensably important function of NA in H7N9 virus is to promote viral mobility toward/within the infected host through protecting virus particles from entrapment by mucin-rich secretions in the respiratory tract as well as through facilitation of viral egress from infected host cells. Thus, the typical N9 NA protein structure contains conserved residues of active sites that contact neuraminic acid as well as residues that provide basic requirements for catalysis, including calcium ion-binding sites, by interacting with both water molecules and backbone carbonyl oxygen atoms at specific sites (293/297/324/347 aa) based on N2 numbering [33]. In this study, the NA S171N mutation had already occurred once with 0.15% of all samples of NA sequence in the strain (A/goose/Jiangsu/J1111/2014) in China. Nevertheless, in addition to G335S (Gly335Ser,AH-H7N9) and N171S (Asn171Ser, AH-H7N9) in this study, which was located within an adjacent area as previously reported, the possibility of other specific mutations that may be critical to conformation stabilization within the active sites of NA proteins cannot be ruled out. The resolved structures of neuraminidase from A/Shanghai/2/2013 (H7N9) influenza virus showed that the NA-S171N mutation contributes to NA tetramerization interfaces and binding of small ligand(s). The NA G335S mutation is involved in viral oligomerization interfaces and binding of small ligand(s) as well as antibody recognition sites as shown in Fig. 6a–d.

Apart from mutations occurring in the HA and NA genes, Shanghai/2 passaging strains also had some mutations of interest, including PA (M86V, Y161A), NP (A284T) and NS1 (D101N). The SH-NS1 D101N and E208K mutations are not found in sequences used to derive mutation statistics. The NS1 D101N position mutated except for NS1-E208K is involved in binding of small ligand(s) or host protein(s), or viral oligomerization interfaces, as found in resolved structures of proteins from related strains. The PA M86V and PA Y161A mutations have been reported to be involved in viral oligomerization interfaces, as shown in Additional file 4: Fig. 4a–b. NA N69I is not found in sequences used to derive mutation statistics. The NA position equivalent to this mutation is involved in viral oligomerization interfaces (Additional file 4: Fig. 4C) Mutation NP M352I and Anhui-NP A373T already occurred many times The NP A373T position but NP M352I is involved in viral oligomerization interfaces, a T-cell epitope presented by MHC molecules as shown in Fig. 4D.

Nevertheless, Shanghai/2-H7N9 strains PB1 (S515F,P596S), PB2 (I185S), NP (M352I) and NS (E208K), and AH-H7N9 strains PA (A343T, A347T and E349T), PB1 (D76N and V451E), PB2 (D87G) are not found in sequences used to derive mutation statistics, thus requiring further investigation of the underlying mechanisms by reverse genetics in H7N9 viruses.

## Conclusion

In this study, we identified quite a few human-adapted variants of AH-H7N9 and SH-H7N9 by serial passaging in MDCK cells. Our results revealed that some of these variants displayed higher variability in replicative capability in both subtypes with multiple amino acid mutations in the H7 and N9 proteins, which was further demonstrated by enhanced receptor affinity to 3′SL/6′SL and NA activity, as well as increased virulence in those strains.

## Supplementary information

**Additional file 1: Figure 1.** Long-term serial passaging of virus strains.

**Additional file 2: Figure 2.** Absolute RNA amounts in viruses from supernatants or cells, quantified by quantitative PCR for the first- or second-generation of viruses in human lung cancer A549 cells..

**Additional file 3: Figure 3.** Illustration of the five rounds of H7N9 outbreak that occurred in China from 2013 to 2017

**Additional file 4: Figure 4.** Crystal structure of representative PA, NP and NA. (A) Shanghai2 PA M86V mutation position (red atoms) on viral chain B (yellow backbone), within 5 A from oligomeric subunit chain A (blue backbone); (B) Shanghai2 PA Y161A mutation position (red atoms) on viral chain C (yellow backbone), within 5 A from oligomeric subunit chain A (blue backbone). (C) Anhui/2 NP A373T mutation position (red atoms) corresponds to position 8 on viral chain F (yellow backbone); (D) Wild Anhui/1 NA-M26I (compared with wild Shanghai2) mutation position (red atoms) corresponds to position 84 on viral chain A (yellow backbone), within 5 A from ligand NAG (pink atoms).

## Data Availability

All data generated or analyzed during this study are included in this published article and its supplementary information files.

## Abbreviations

AH-H7N9: A/Anhui/1/2013
SH-H7N9: A/Shanghai/2/2013
HA7: hemagglutinin 7
IAV: influenza type A virus
NA: neuraminidase
HPAI: highly pathogenic avian influenza
LPAI: low pathogenicity avian influenza
SA: sialic acid
TCID_50_: tissue culture infective dose
MDCK: Madin-Darby canine kidney cells
CPE: cytopathic effect
