# Supplementary figures and images for: Identifying novel amino acid substitutions of hemagglutinin involved in virulence enhancement in H7N9 virus strains
Source: Virol J. 2021 Jan 11;18:14. doi: 10.1186/s12985-020-01464-1 (PMC7798331; doi:10.1186/s12985-020-01464-1)

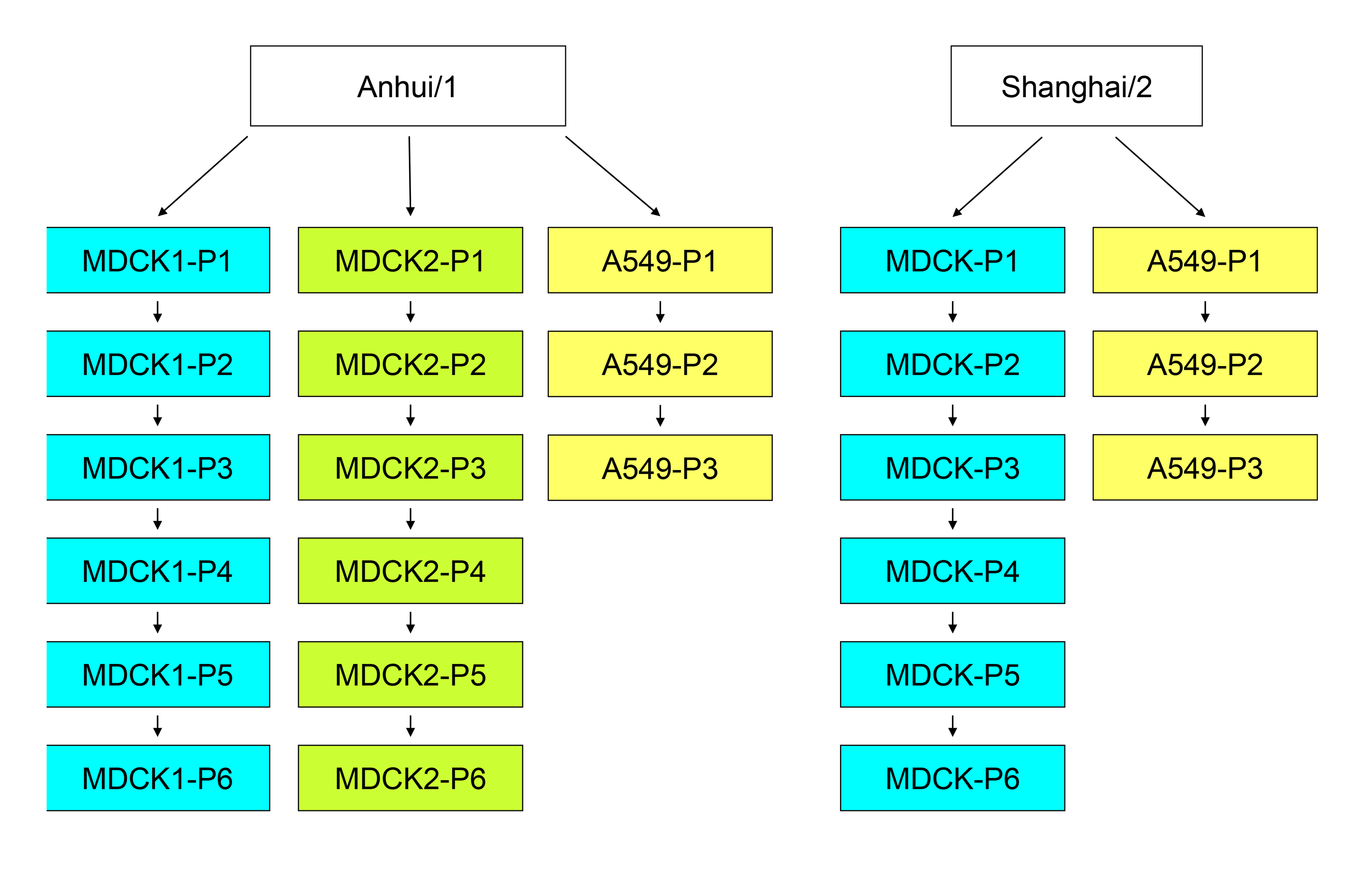

Supplement: Supplementary file 1 — Additional file 1: Figure 1. Long-term serial passaging of virus strains. [file 12985_2020_1464_MOESM1_ESM.tif]

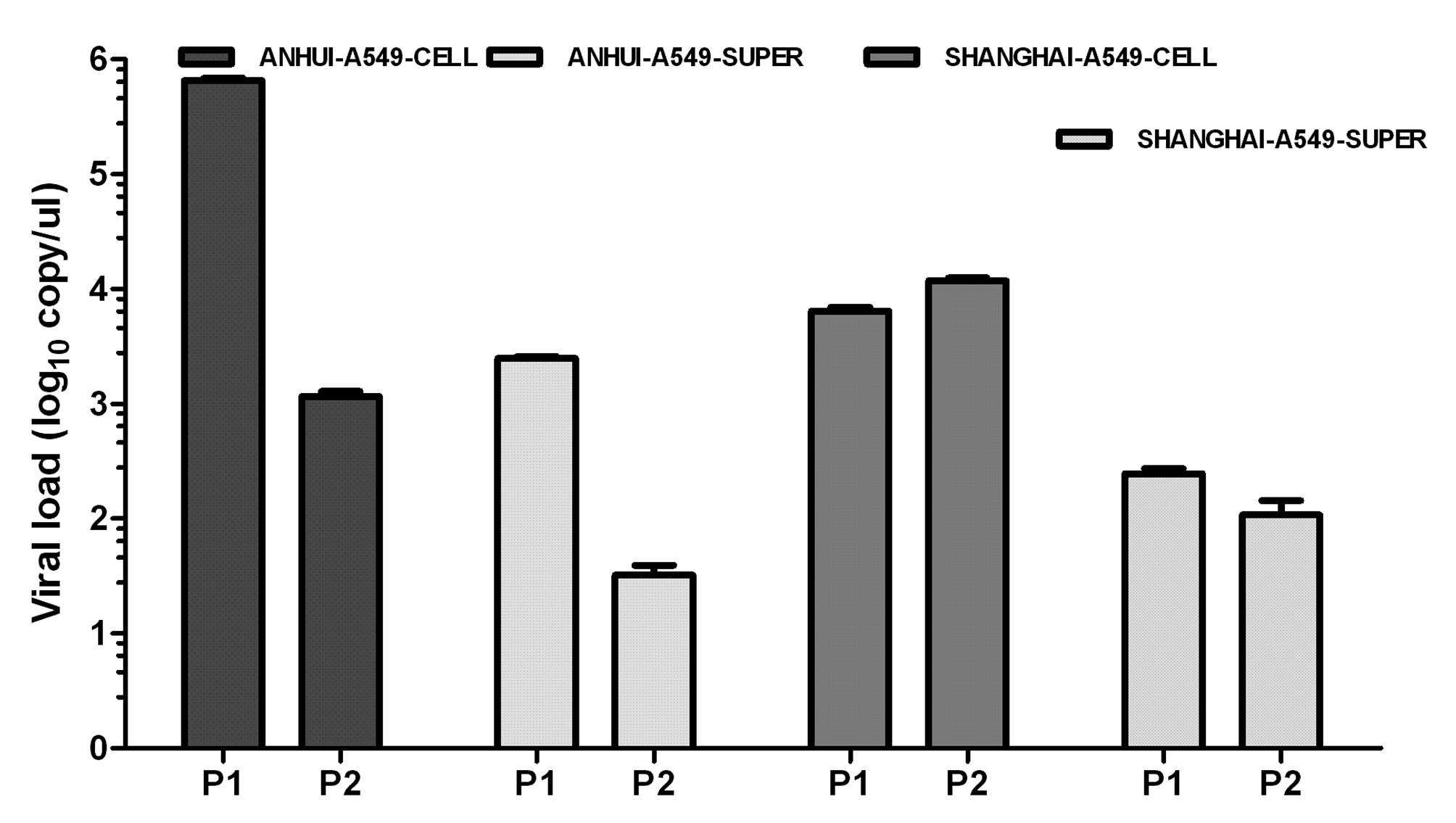

Supplement: Supplementary file 2 — Additional file 2: Figure 2. Absolute RNA amounts in viruses from supernatants or cells, quantified by quantitative PCR for the first- or second-generation of viruses in human lung cancer A549 cells.. [file 12985_2020_1464_MOESM2_ESM.tif]

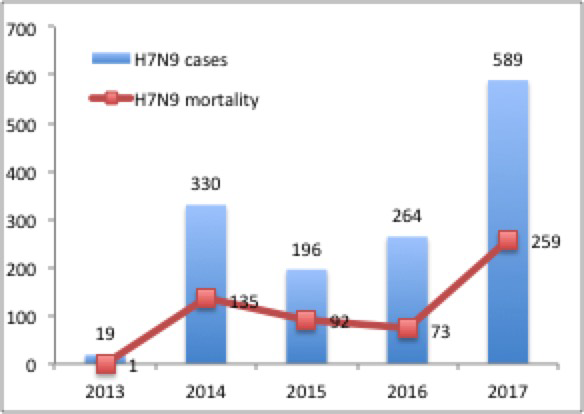

Supplement: Supplementary file 3 — Additional file 3: Figure 3. Illustration of the five rounds of H7N9 outbreak that occurred in China from 2013 to 2017 [file 12985_2020_1464_MOESM3_ESM.tif]

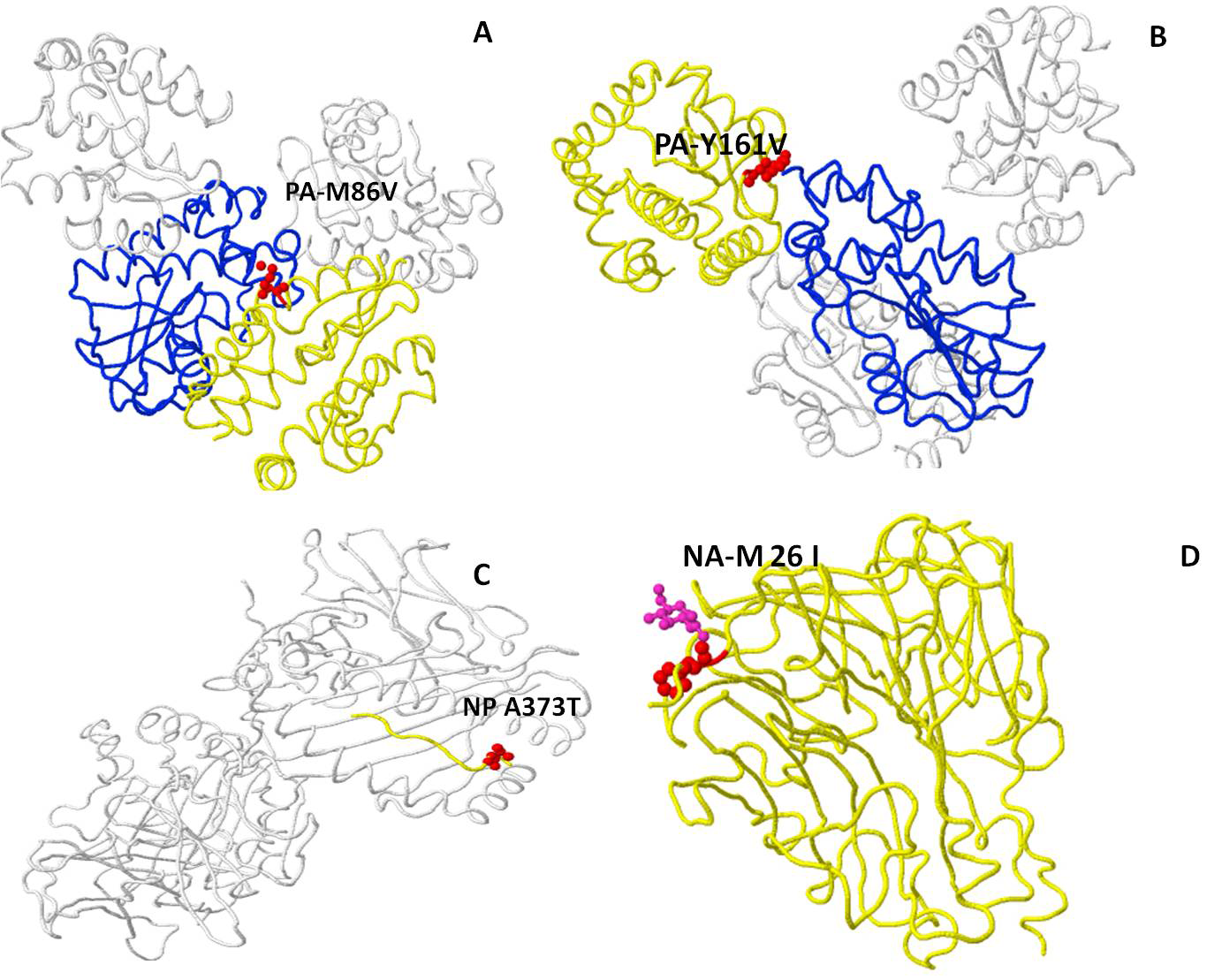

Supplement: Supplementary file 4 — Additional file 4: Figure 4. Crystal structure of representative PA, NP and NA. (A) Shanghai2 PA M86V mutation position (red atoms) on viral chain B (yellow backbone), within 5 A from oligomeric subunit chain A (blue backbone); (B) Shanghai2 PA Y161A mutation position (red atoms) on viral chain C (yellow backbone), within 5 A from oligomeric subunit chain A (blue backbone). (C) Anhui/2 NP A373T mutation position (red atoms) corresponds to position 8 on viral chain F (yellow backbone); (D) Wild Anhui/1 NA-M26I (compared with wild Shanghai2) mutation position (red atoms) corresponds to position 84 on viral chain A (yellow backbone), within 5 A from ligand NAG (pink atoms). [file 12985_2020_1464_MOESM4_ESM.tif]
